# Toxicity assessment and analgesic activity investigation of aqueous acetone extracts of *Sida acuta * Burn f *.* and *Sida cordifolia * L. (Malvaceae), medicinal plants of Burkina Faso

**DOI:** 10.1186/1472-6882-12-120

**Published:** 2012-08-11

**Authors:** Kiessoun Konaté, Imaël Henri Nestor Bassolé, Adama Hilou, Raïssa RR Aworet-Samseny, Alain Souza, Nicolas Barro, Mamoudou H Dicko, Jacques Y Datté, Bertrand M’Batchi

**Affiliations:** 1 Laboratory of Biochemistry and Applied Chemistry, University of Ouagadougou, 09 P.O.Box: 848, Ouagadougou 09, Burkina Faso; 2 Laboratory of Food Biochemistry, Enzymology, Biotechnology and Bioinformatic, University of Ouagadougou, 03 P.O.Box: 848, Ouagadougou 03, Burkina Faso; 3 Institut Pharmacopoeia and Traditional Medicine, National Center for Scientific and Technological Research, P.O.Box: 1156, Libreville, Gabon; 4 Laboratory of Animal Physiology, Electrophysiology and Pharmacology, Faculty of Sciences, University of Science and Technology of Masuku, Franceville, Gabon; 5 Laboratory of Biochemistry and Molecular Genetics Microbial, University of Ouagadougou, 03 P.O.Box: 7131, Ouagadougou 03, Burkina Faso; 6 Laboratory of Nutrition and Pharmacology, Faculty of Biosciences, University of Cocody, Abidjan 22 P.O.Box: 582, Abidjan, Cote d’Ivoire

## Abstract

**Background:**

*Sida acuta * Burn f. and *Sida cordifolia * L. (Malvaceae) are traditionally used in Burkina Faso to treat several ailments, mainly pains, including abdominal infections and associated diseases. Despite the extensive use of these plants in traditional health care, literature provides little information regarding their toxicity and the pharmacology. This work was therefore designed to investigate the toxicological effects of aqueous acetone extracts of *Sida acuta * Burn f. and *Sida cordifolia * L. Furthermore, their analgesic capacity was assessed, in order to assess the efficiency of the traditional use of these two medicinal plants from Burkina Faso.

**Method:**

For acute toxicity test, mice were injected different doses of each extract by intraperitoneal route and the LD50 values were determined. For the subchronic toxicity evaluation, Wistar albinos rats were treated by gavage during 28 days at different doses of aqueous acetone extracts and then haematological and biochemical parameters were determined. The analgesic effect was evaluated in mice by the acetic-acid writhing test and by the formalin test.

**Results:**

For the acute toxicity test, the LD50 values of 3.2 g/kg and 3.4 g/kg respectively for *S. acuta * Burn f. and *S. cordifolia * L. were obtained. Concerning the haematological and biochemical parameters, data varied widely (increase or decrease) according to dose of extracts and weight of rats and did not show clinical correlations. The extracts have produced significant analgesic effects by the acetic acid writhing test and by the hot plate method (p <0.05) and a dose-dependent inhibition was observed.

**Conclusion:**

The overall results of this study may justify the traditional uses of *S. acuta * and *S. cordifolia *.

## Background

Indigenous medicinal plants were and are still one of the sources of modern medicines [1]. Moreover the trend of using phytotherapy as alternative medicine has increased the interest for the tropical plants’ pharmacognosy [2].

In Africa, especially in Burkina Faso, medicinal plants still play an important role in health care of an important portion of the population. This is because they are cheap, are locally available and efficient. Generally, the effects of medicinal plants are attributed to their content in active chemicals [3]. In developing countries there is a general belief among the consumers that the use of medicinal plants is always safe because they are “natural”. However, evidences suggest otherwise and some studies suggest that some of the herbs can be associated with health hazards. Medicinal plants can contain many active chemical compounds and also other substances of great complexity like mucilages, polyphenols, polysaccharides, etc. [3]. That may modulate and modify the effects of any “active principles”. Thus, some herbal remedies can be toxic or can act either as agonists or antagonists of the active principles. Therefore, the study of toxicity is an essential prerequisite for the efficiency assessment of plant extracts.

An ethnobotanical investigation in the central region of Burkina Faso has shown that many species are traditionally used to treat various kinds of pain diseases. Among such plants, *S. acuta * Burn f. and *S. cordifolia * L. (Malvaceae) are the most frequently and widely used. These plants are used to treat infectious diseases in children such as malaria, fever, pain, variola, and also have antibacterial, anti-inflammatory, analgesic and hepatoprotective properties [3,4]. In most cases, the drugs are administrated over a long period of time and without any proper monitoring of the dosage.

Previous data showed that aqueous acetone extracts of *S. acuta * Burn f. and *S. cordifolia * L. contain saponosides, coumarins, steroids, phenolic compounds and alkaloids. In addition, their extracts have showed good antioxidant and anti-inflammatory activities [5]. Despite their interesting biochemical features, the toxicology profile and the analgesic properties of these extracts are lacking. The aim of this contribution is to evaluate the toxicity of aqueous acetone extracts of *S. acuta * Burn f. and *S. cordifolia * L., as well as their analgesic properties.

## Methods

### Identification of the plant materials

*S. acuta * Burn f. and *S. cordifolia * L. were collected in August 2008 in Gampela, 25 Km east of Ouagadougou, capital of Burkina Faso. The plants were identified in the Laboratory of Biology and Ecology, University of Ouagadougou, where a voucher specimen was deposited.

### Preparation of extracts

Fifty grams of powdered plant materials (dried in laboratory condition) was extracted with 500 ml of acetone 80% (400 ml acetone + 100 ml water) for 24 h under mechanic agitation (SM 25 shaker, Edmund BÜHLER, Germany) at room temperature. After filtration, acetone was removed under reduced pressure in a rotary evaporator (BÜCHI, Rotavopor R-200, Switzeland) at approximately 40°C and freeze-dried (Telstar Cryodos 50 freeze-dryer). The extracts were weighed before packing in waterproof plastic flasks and stored at 4°C until use. The yields of the extractions were measured with precision balance (ADVENTURER). For yield of the extracts (mass of extract x 100/mass of powder), we obtained 13.42% for *S. acuta * and 9.45% for *S. cordifolia. *

### Animals handling

Swiss NMRI mice (25–30 g) and adult albinos Wistar rats (160-165 g) of both sexes were used for this study. All animals were housed in cages under controlled conditions of 12-h light/and 12 h without light and 25°C. They received pellets of food enriched with 20% protein and water ad libitum. They were deprived of food for 15 h (but with access to drinking water) and weighed before the experiments. Experiments on the animals were performed according to the protocols already approved by the Institute of Health Sciences Research/University of Ouagadougou (Burkina Faso) and met the international standards for animal study [6].

### Toxicity studies

#### For acute toxicity

Swiss mice (male and female) were randomly divided into 7 groups (1 control group and 6 treated groups) of 6 animals (3 males and 3 females). The control group received water containing 10% dimethylsulfoxide (DMSO) administered intraperitoneally. The aqueous acetone extracts of *S. acuta* Burn f. and *S. cordifolia * L., suspended in 10% DMSO were administered intraperitoneally at doses of 1; 2; 2.5; 3; 4; 5 and 6 g/kg. The general behaviour of the mice was observed for 120 min after the treatment. The animals were observed for morbidity and mortality once a day for 14 days. The number of survivors after the 14 days period was noted. The toxicological effect was assessed on the basis of mortality for 14 days, which was expressed by the median lethal dose value (Lethal Dose 50 or LD50) estimated from the regression of log-probit mortality rate [7].

#### For subchronic toxicity study

Wistar rats were divided into 5 groups of 6 animals (3 males and 3 females) for each type of extract. The first groups served as control, and they received water containing DMSO 10%. The second, the third and the fourth group of rats received daily and orally (gavage) for 28 days respectively 75, 100, and 200 mg/kg of each of two type of extract (suspended in 10% of DMSO). Body weight was weekly taken, and the animals were daily observed to detect any signs of abnormalities throughout the study period. At the end of the 28 days period, the animals were deprived of food for 15 h. Then blood samples were collected by cardiac puncture for biochemical and hematological tests, and selected organs were carefully removed and weighed.

#### Collection of blood samples

Blood samples were collected by cardiac puncture in three tubes for haematology, glucose and serum analysis. The blood samples with heparin and those without anticoagulant were centrifuged at 3000 rpm for 5 min to obtain plasma and serum respectively. Plasma was used to determine glucose [8,9] and the serum for other biochemical parameters such as aspartate aminotransferase (AST) [10], alanine aminotransferase (ALT) [11], alkaline phosphatase (ALP) [12,13], total bilirubin and direct bilirubin [14], triglycerides [15], total cholesterol [16]. All these biochemical parameters were measured with a laboratory automat (Selectra XL Vital Scientific, Elitech Group Company).

Hematological analyses were performed on the whole blood using an automatic counter (Mindray Auto hematology Analyser BC-5500) to evaluate the following parameters: total red blood cells (RBC), hemoglobin, hematocrit, platelet count, leukocytes (WBC), neutrophilis, basophilis, eosinophils, lymphocytes, monocytes, MCV (mean corpuscular volume), MCH (mean corpuscular hemoglobin ) and MCHC (mean corpuscular hemoglobin concentration).

### Test of analgesic properties

#### Test of writhing induced by acetic acid

The analgesic properties of the extracts were analyzed using the writhing (induced by acetic acid) on a mice model [17]. Nociception was induced by an intraperitoneal injection of 0.6% acetic acid with a dosage of 10 ml/kg of body weight. For each extract five groups of six mice were formed. Each animal of group I received 10 ml/kg of body weight of the vehicle (10% DMSO in water), and those from group II received 100 mg/kg of body weight of paracetamol. Each animal from group III, group IV and group V were orally treated with 200; 400 and 600 mg/kg of body weight doses of extracts (dissolved in 10% DMSO) respectively, one hour before acetic acid injection. The number of writhings occurring between 5 and 20 min after acetic acid injection was recorded. The analgesic effect was expressed as the percentage reduction of writhes in treated mice compared to those in the control. The percentage inhibition was calculated using the formula below:

$\left(\%\right)\text{inhibition}=\left(\text{A}-\text{B/A}\right)\times 100$, where A is mean for the control group and B is mean for the treated group.

#### The formalin-induced nociception

The analgesic effect of *S. acuta * Burn f. and *S. cordifolia * L. was also evaluated using the method of paw licking, induced by formalin [18]. The animals were divided into five groups of six mice. Each animal of Group I received 10 ml/kg of body weight of the vehicle solution (10% DMSO in water) while those from group II received 100 mg/kg of body weight of paracetamol. The animals from the groups III, IV and V received orally respectively 200; 400 and 600 mg/kg of body weight doses of extracts (suspended in 10% DMSO). One hour after drug administration, 20 μl of formalin (2.5% in normal saline) was injected into the plantar surface of the left hind paw of mice. The time spent in licking the injected paw was recorded and expressed as the total licking time in early phase (0 to 5 min after formalin injection) and late phase (15 to 30 min). The percentage inhibition was calculated as above.

### Statistical analyses

Data were expressed as Mean ± Standard deviation (SD) of six experiments (n = 6). Results were analyzed by one-way ANOVA followed by Dunnett’s t-test using Prism 4 software. The level of significance was considered at p ≤0.05.

## Results

### Acute toxicity study of the plant extracts

For the acute toxicity study in mice, the following values of LD50 were obtained: 3.2 g/kg and 3.4 g/kg respectively for *S. acuta * Burn f. and *S.cordifolia * L.

### Concerning subchronic toxicity study in rats

#### Body weights

**Figure 1:**
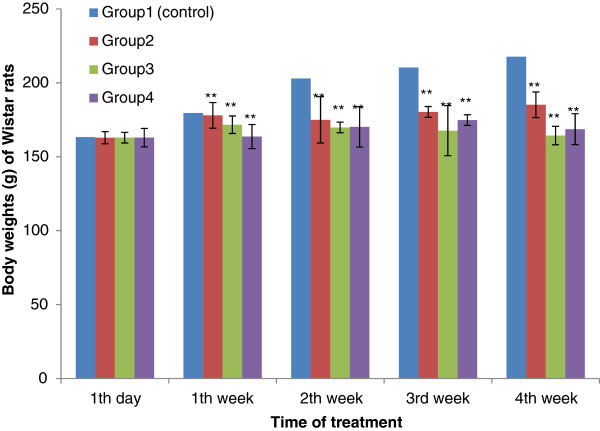
**Effect of Aqueous Acetone Extract of *Sida acuta* on body weights (g) of Wistar rats with the time of treatment.** Values are mean ± S.E.M. (n = 6 in each group) one-way ANOVA followed by Dunnett’s *t*- test: Compare all vs. control: p>0.05, *p <0.05, **p <0.01 compared with control. Group 1: control, rats received 10% DMSO. Group 2: rats received 10% DMSO with extract (75 mg/kg body weight). Group 3: rats received 10% DMSO with extract (100 mg/kg body weight). Group 4: rats received 10% DMSO with extract (200 mg/kg body weight).

**Figure 2:**
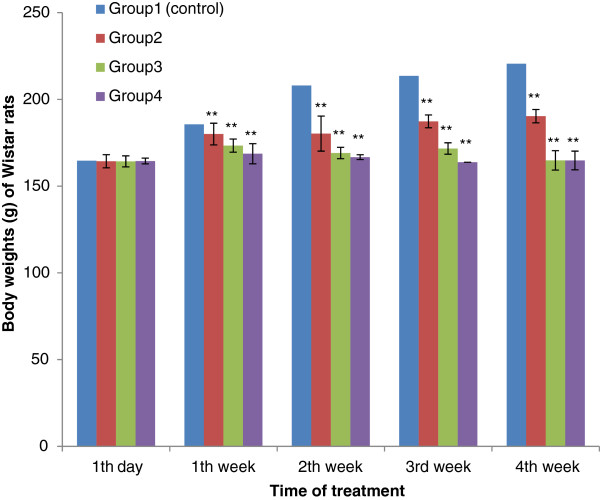
**Effect of Aqueous Acetone Extract of *Sida cordifolia*  on body weights (g) of Wistar rats with the time of treatment.** Values are mean ± S.E.M. (n = 6 in each group) one-way ANOVA followed by Dunnett’s *t*- test: Compare all vs. control: p>0.05, *p <0.05, **p <0.01 compared with control. Group 1: control, rats received 10% DMSO. Group 2: rats received 10% DMSO with extract (75 mg/kg body weight). Group 3: rats received 10% DMSO with extract (100 mg/kg body weight). Group 4: rats received 10% DMSO with extract (200 mg/kg body weight).

No significant difference in body-weight gain between control group and the test groups was observed in the first days of treatment (p>0.05). However, with four weeks, a significant decrease in body weight was noticed between the test groups and the control groups (p <0.01). The results are summarised in Figure1 and Figure2.

#### Relative organ weights

**Figure 3:**
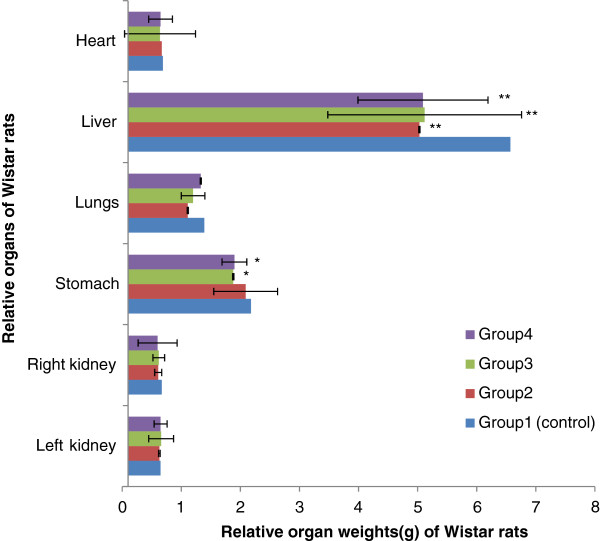
** Effect of Aqueous Acetone Extract of *Sida acuta* on relative organ weights (g) of rats.** Values are mean ± S.E.M. (n = 6 in each group) one-way ANOVA followed by Dunnett’s *t*- test: Compare all vs. control: p>0.05, *p <0.05, **p <0.01 compared with control. Group 1: control, rats received 10% DMSO. Group 2: rats received 10% DMSO with extract (75 mg/kg body weight). Group 3: rats received 10% DMSO with extract (100 mg/kg body weight). Group 4: rats received 10% DMSO with extract (200 mg/kg body weight).

**Figure 4:**
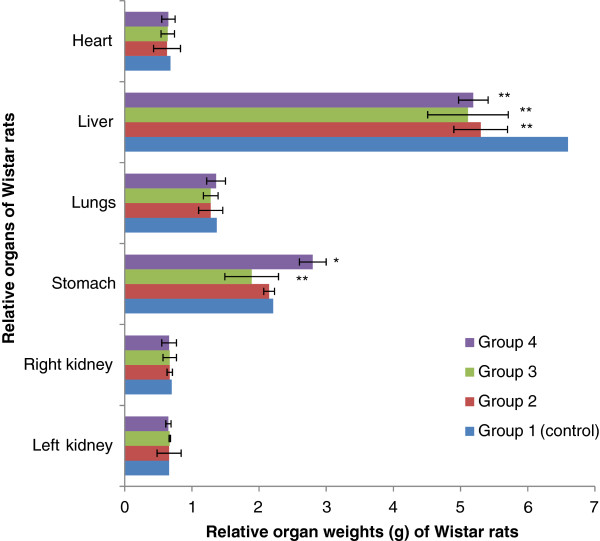
** Effect of Aqueous Acetone Extract of *Sida cordifolia * on relative organ weights (g) of rats.** Values are mean ± S.E.M. (n = 6 in each group) one-way ANOVA followed by Dunnett’s *t*- test: Compare all vs. control: p>0.05, *p <0.05, **p <0.01 compared with control. Group 1: control, rats received 10% DMSO. Group 2: rats received 10% DMSO with extract (75 mg/kg body weight). Group 3: rats received 10% DMSO with extract (100 mg/kg body weight). Group 4: rats received 10% DMSO with extract (200 mg/kg body weight).

Figure3 and Figure4 show the effects of extracts on weight of some vital body organs of rats. No significant difference for kidneys, stomach, lungs and heart (p>0.05); but we noticed a significant decrease about the liver weight between the treated groups and the control group (p <0.05).

#### Haematological analysis

The effects of extracts on the haematological parameters are summarised in Table1. There is a significant decrease for monocytes, basophils, haemoglobin, haematocrit and MCV between the control group (10% DMSO) and the treated groups (p <0.05 or p <0.01).

**Table 1:** **Effect of Aqueous Acetone Extracts of *Sida acuta * and *Sida cordifolia * on the haematological parameters on whole blood of rats**

**Haematological parameters**	**Group 1**	**Group 2**	**Group 3**	**Group 4**
*Sida acuta* Aqueous Acetone Extract				
WBC(10^3^/μl)	12.43 ± 3.20	12.19 ± 0.10^ns^	11.69 ± 1.20^ns^	11.77 ± 0.11^ns^
RBC (10^6^/μl)	7.17 ± 1.18	7.23 ± 2.36^ns^	7.13 ± 1.03^ns^	7.08 ± 0.88^ns^
Eosinophil (%)	2.18 ± 0.68	2.21 ± 1.08^ns^	2.00 ± 2.20^ns^	2.11 ± 4.53^ns^
Lymphocyte (%)	78.11 ± 0.24	77.85 ± 1.57^ns^	77.69 ± 1.43^ns^	78.00 ± 2.21^ns^
Neutrophil (%)	25.89 ± 0.09	25.78 ± 0.31^ns^	25.59 ± 1.43^ns^	25.81 ± 1.21^ns^
Monocyte (%)	1.57 ± 0.24	1.02 ± 1.20*	1.00 ± 1.43*	0.91 ± 1.21*
Basophil (%)	0.17 ± 0.09	0.13 ± 0.31^ns^	0.11 ± 0.43**	0.12 ± 0.22^ns^
Haemoglobin (g/dl)	15.23 ± 1.13	14.69 ± 0.15^ns^	14.21 ± 0.63*	14.11 ± 0.77 *
Haematocrit (%)	43.46 ± 2.20	44.00 ± 1.21^ns^	42.43 ± 3.20*	40.61 ± 1.2**
MCV (μm^3^)	57.00 ± 0.48	56.48 ± 1.28^ns^	55.35 ± 0.15*	54.79 ± 2.30**
MCH (pg)	18.87 ± 1.20	18.62 ± 0.43^ns^	18.90 ± 1.22^ns^	18.49 ± 0.67^ns^
MCHC (g/dl)	33.78 ± 0.11	33.35 ± 2.22^ns^	33.00 ± 0.40^ns^	33.13 ± 1.22^ns^
Platelet (x10^3^/μl)	967.42 ± 4.12	961.11 ± 5.10 ^ns^	953.22 ± 2.35 ^ns^	964.00 ± 1.10 ^ns^
*Sida cordifolia* Aqueous Acetone Extract				
WBC(10^3^/μl)	12.21 ± 2.21	12.08 ± 0.54^ns^	12.00 ± 1.10^ns^	12.02 ± 1.22^ns^
RBC (10^6^/μl)	7.29 ± 0.21	7.45 ± 0.15^ns^	7.22 ± 0.59^ns^	7.10 ± 0.27^ns^
Eosinophil (%)	2.23 ± 1.03	2.14 ± 0.56^ns^	2.00 ± 0.53^ns^	2.17 ± 4.53^ns^
Lymphocyte (%)	78. 31 ± 1.03	77.60 ± 0.88^ns^	77.62 ± 1.20^ns^	76.89 ± 0.22*
Neutrophil (%)	26.13 ± 0.68	26.00 ± 1.08^ns^	25.78 ± 2.15^ns^	26.13 ± 2.33^ns^
Monocyte (%)	2.01 ± 0.09	0.76 ± 0.31*	0.82 ± 1.43*	0.91 ± 1.21*
Basophil (%)	0.2 ± 0.24	0.17 ± 0.10^ns^	0.17 ± 1.25^ns^	0.13 ± 0.36*
Haemoglobin (g/dl)	15.02 ± 1.43	14.40 ± 0.31^ns^	14.23 ± 0.54^ns^	14.75 ± 1.21^ns^
Haematocrit (%)	42.83 ± 2.21	42.60 ± 1.10^ns^	41.67 ± 0.33*	42.00 ± 0.22^ns^
MCV (μm^3^)	56.78 ± 0.15	56.62 ± 0.34^ns^	56.41 ± 2.10^ns^	56.80 ± 0.33^ns^
MCH (pg)	19.61 ± 0.24	19.57 ± 0.22^ns^	19.07 ± 1.20^ns^	19.55 ± 0.36^ns^
MCHC (g/dl)	33.26 ± 0.1	33.51 ± 0.54^ns^	33.37 ± 2.21^ns^	33.00 ± 0.2^ns^
Platelet (x10^3^/μl)	960.11 ± 0.33	957.23 ± 0.67^ns^	963.47 ± 1.63^ns^	966.40 ± 0.67^ns^

*WBC*: leucocyte count; *RBC*: erythrocyte count; *MCV*: mean corpuscular volume; *MCH*: mean corpuscular haemoglobin; *MCHC*: mean corpuscular haemoglobin concentration.

Values are mean ± S.E.M. (n = 6) one-way ANOVA followed by Dunnett’s *t*- test: Compare all vs. control: p>0.05, *p <0.05, **p <0.01 compared with control

Group 1: control, rats received 10% DMSO.

Group 2: rats received 10% DMSO with extract (75 mg/kg body weight).

Group 3: rats received 10% DMSO with extract (100 mg/kg body weight).

Group 4: rats received 10% DMSO with extract (200 mg/kg body weight).

#### Biochemical analyses

Table2 shows the effects of extracts on the biochemical parameters. For certain biochemical parameters (AST, ALT, ALP) one observed a significant increase (p <0.01); however for the other biochemical parameters (glucose, creatinine, urea nitrogen, triglycerides, total bilirubin and direct bilirubin), there is a significant decrease between the control group (10% DMSO) and the other treated groups (p <0.05 or p <0.01).

**Table 2:** **Effect of Aqueous Acetone Extracts of *Sida acuta * and *Sida cordifolia*  on the biochemical parameters in the plasma and the serum of rats**

**Biochemical Parameters**	**Group 1**	**Group 2**	**Group 3**	**Group 4**
*Sida acuta * Aqueous Acetone Extract				
Glucose (mmol/l)	6.21 ± 1.12	4.28 ± 0.022**	3.17 ± 0.11**	3.39 ± 0.01**
Uric acid (mmol/l)	0.17 ± 1.18	0.12 ± 0.02^ns^	0.12 ± 0.01^ns^	0.14 ± 0.33^ns^
Urea nitrogen (mmol/l)	9.64 ± 0.1	9.41 ± 0.22^ns^	9.44 ± 0.03^ns^	9.46 ± 0.02^ns^
Creatinine (mmol/l)	0.053 ± 0.01	0.047 ± 0.001^ns^	0.048 ± 0.003*	0.051 ± 0.01*
AST (UI/l)	79.5 ± 2.20	100 ± 0.54**	103.00 ± 3.20**	105.33 ± 1.11**
ALT (UI/l)	39.5 ± 5.40	81.00 ± 13.18**	77.5 ± 1.10**	89.00 ± 0.67**
ALP (UI/l)	71.5 ± 2.00	106.13 ± 2.40**	109.22 ± 1.20**	110 ± 2.18**
Triglycerides (mmol/l)	0.80 ± 0.06	0.70 ± 0.03^ns^	0.73 ± 0.03*	0.74 ± 0.01*
Total cholesterol (mmol/l)	2.15 ± 0.33	2.03 ± 0.01^ns^	2.07 ± 0.02^ns^	2.08 ± 0.01^ns^
Total bilirubin (mmol/l)	0.14 ± 0.01	0.12 ± 0.03^ns^	0.12 ± 0.01^ns^	0.13 ± 0.02^ns^
Direct bilirubin (mmol/l)	0.0013 ± 0.11	0.0012 ± 1.10^ns^	0.0011 ± 0.01^ns^	0.0013 ± 0.01^ns^
*Sida cordifolia * Aqueous Acetone Extract				
Glucose (mmol/l)	6.30 ± 0.10	4.41 ± 033**	3.51 ± 0.11**	3.60 ± 0.67**
Uric acid (mmol/l)	0.173 ± 1.10	0.170 ± 0.21^ns^	0.169 ± 0.33^ns^	0.171 ± 0.22^ns^
Urea nitrogen (mmol/l)	9.61 ± 0.33	9.49 ± 2.20^ns^	9.55 ± 0.67^ns^	9.58 ± 1.21*
Creatinine (mmol/l)	0.053 ± 0.11	0.034 ± 1.22^ns^	0.044 ± 0.04^ns^	0.047 ± 0.01*
AST (UI/l)	84.12 ± 2.20	97.67 ± 3.10**	100.60 ± 1.21**	102.33 ± 0.33**
ALT (UI/l)	41.62 ± 11.61	51.21 ± 5.44**	80.12 ± 13.67**	89.55 ± 0.54**
ALP (UI/l)	73.51 ± 1.74	88.12 ± 2.21**	105.33 ± 0.33**	107.33 ± 1.67**
Triglycerides (mmol/l)	0.58 ± 1.15	0.57 ± 0.33^ns^	0.55 ± 0.67*	0.57 ± 0.54*
Total cholesterol (mmol/l)	2.10 ± 0.33	1.62 ± 0.15^ns^	1.85 ± 0.01^ns^	2.01 ± 0.67^ns^
Total bilirubin (mmol/l)	0.2 ± 0.071	0.121 ± 0.33*	0.130 ± 0.033^ns^	0.159 ± 0.067**
Direct bilirubin (mmol/l)	0.0034 ± 0.67	0.0024 ± 0.10^ns^	0.0026 ± 0.11^ns^	0.0029 ± 0.67*

*AST*: aspartate aminotransferase; *ALT*: alanine aminotransferase; *ALP*: alkaline phosphatase.

Values are mean ± S.E.M. (n = 6) one-way ANOVA followed by Dunnett’s *t*- test: Compare all vs. control:

p>0.05,*p <0.05, **p <0.01 compared with control.

Group 1: control, rats received 10% DMSO

Group 2: rats received 10% DMSO with extract (75 mg/kg body weight).

Group 3: rats received 10% DMSO with extract (100 mg/kg body weight).

Group 4: rats received 10% DMSO with extract (200 mg/kg body weight).

### Analgesic properties

#### Acetic-acid writhing test

**Figure 5:**
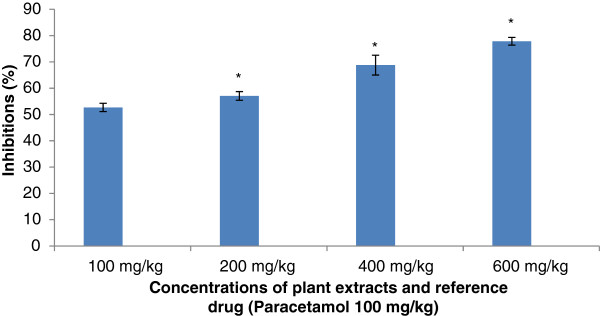
**Effect of Aqueous Acetone Extract of *Sida acuta*  on writhing-induced by acetic acid.** Values are mean ± S.E.M. (n = 6 in each group) one-way ANOVA followed by Dunnett’s *t*- test: Compare all vs. control: *p <0.05 compared with control.

**Figure 6:**
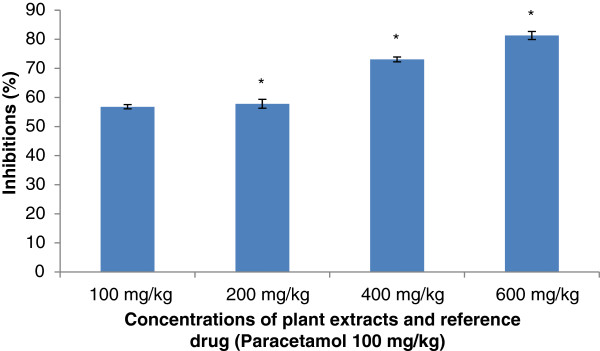
**Effect of Aqueous Acetone Extract of * Sida cordifolia * on writhing-induced by acetic acid.** Values are mean ± S.E.M. (n = 6 in each group) one-way ANOVA followed by Dunnett’s *t*- test: Compare all vs. control: *p <0.05 compared with control.

The extracts have effectively reduced the number of abdominal muscle contractions induced by 0.6% acetic acid solution. The extracts produced significant inhibition of writhing induced by acetic acid. The inhibition was dose-dependent. The results are presented in Figure5 and Figure6. However the extract of *S. cordifolia * has produced higher inhibition values comparatively to the extract of *S. acuta *.

#### Formalin-induced nociception

**Figure 7:**
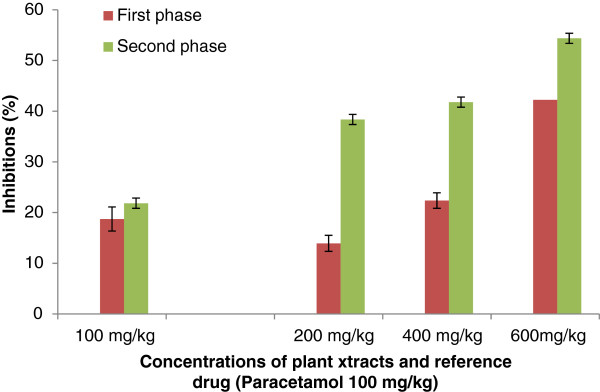
**Effect of Aqueous Acetone Extract of *Sida cordifolia*  on licking the hind paw-induced by formalin injection.** Values are mean ± S.E.M. (n = 6 in each group) one-way ANOVA followed by Dunnett’s *t*- test: Compare all vs. control: p <0.01.

**Figure 8:**
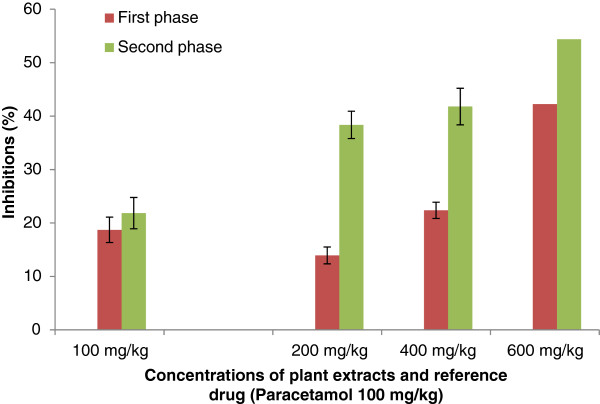
**Effect of Aqueous Acetone Extract of *Sida acuta*  on licking the hind paw-induced by formalin injection.** Values are mean ± S.E.M. (n = 6 in each group) one-way ANOVA followed by Dunnett’s *t*- test: Compare all vs. control: p <0.01.

For the formalin-induced nociception; it can also be noticed that the extracts have significantly inhibited the inflammation induced by formalin. The extracts have inhibited the early phase (0 to 5 min) and the second phase (15 to 30) of inflammation induced by formalin. The inhibition was also dose-dependent. The extract of *S. cordifolia* has also produced higher inhibition than *S. acuta* (Figure7 and Figure8).

## Discussion

About the acute toxicity, data indicated that the aqueous acetone extracts of *S. acuta * Burn f. and *S. cordifolia * L. can be considered as weakly poisonous. Indeed, the literature reports that the substances with LD50 higher than 5 g/kg b.w., by oral route are regarded as being safe or practically non-toxic [19]. This is an indication that the aqueous acetone extracts of *S. acuta * Burn f. and *S. cordifolia* L., have negligible level of toxicity when administered orally.

Concerning the subchronic toxicity study, no significant difference was observed in body weight gain between control group and the test groups during the first days of treatment. However, after the first week, we noticed a significant difference in body weight between the test groups and the control groups (p <0.01). The weak decrease in body weight observed in the rats treated with the highest doses (100 and 200 mg/kg b.w.) of the extracts, after the first week, may be due to low food intake and its non-optimal utilization. Severe growth depression as a consequence of reduced food intake in rats fed with a high-tannin content diet is well documented [20]. It’s well known that tannins are found in high concentration in the extracts of *S. acuta * Burn f. and *S. cordifolia * L. [5].

The relative organ weight is also an important index of physiological and pathological status in man and animals. The heart, liver, spleen, kidney and lung are the primary organs affected by the metabolic reactions induced by toxicants [21]. Our results suggest that the aqueous acetone extracts are low toxic on these organs.

The analysis of blood parameters is relevant to risk evaluation because the haematological system has a higher predictive value of toxicity in humans when tests involve rodents [22]. The blood features are a good index of physiological and pathological status in man and animals and the parameters usually measured are haemoglobin, total red blood cells (RBC), leukocytes (WBC), MCV, MCH, MCHC, neutrophils, lymphocytes, eosinophils and number of platelets [23]. No significant difference was found in the majority of haematological parameters between the treated and the control groups. This excludes the possibility of anaemia or disturbance linked to erythrocytes. However, a significant decrease was observed between the control group (10% DMSO) and the treated groups for monocytes, basophils, haemoglobin, haematocrit and MCV (p <0.05 or p <0.01). This is an indication of the relative low toxicity of the aqueous acetone extracts on the haemopoetic system. The extracts of *S. acuta * Burn f. and *S. cordifolia * L. contain flavonoids [5] which have been shown to increase vascular integrity and also to act as antihaemorrhagic [24]. No obvious dose response was observed; however, changes observed between blood parameters do not suggest that the aqueous acetone extracts of *S. acuta * Burn f. and *S. cordifolia * L., produced toxicity in the treatment period. These differences may be explained by biological variation which is specific to each rat. [25].

The determination of creatinine, urea and uric acid are markers of kidney function [26]. In this present study, there is a significant (decrease) difference in creatinine and urea amount comparatively to the control group (p <0.05). This was also confirmed by the low variation of kidney weights (right kidney and left kidney).

It was observed dose-dependent elevations of the serum enzymes in the serum enzymes in the treated groups. This indicates hepatocellular damages [27]. Previous studies [28,29] reported that the increase in the activity of these enzymes in the plasma is often observed as a consequence of liver damage. The elevation in AST in all the treated groups and ALP in the highest doses groups suggest that other non-specific tissue damage also occurred because these enzymes have a wider distribution beyond liver [30-32]. However, it can be noticed that there is no too much difference in the levels of AST, ALT and ALP comparatively to the control group (p <0.01 or p <0.05). The results revealed a relationship between these enzymatic markers and liver function and this was demonstrated by the decrease of liver weight.

The treated groups have showed significantly lowered levels of bilirubin, urea and cholesterol. The decrease in bilirubin concentrations may be attributed to the depressant effect of the extracts. Some depressant compounds are known to decrease the concentration of bilirubin.The cholesterol concentration decreased in treated rats and this may indicate hepatocellular damage or malnutrition. It might also be that the extracts possess a hypo-cholesterolemic effect [33]. The serum urea concentration also showed a dose-dependent decrease following extracts administration. Urea is a product of protein metabolism that is excreted in urine and its retention in the body may indicate renal damage [34]. In addition, high levels of urea in control groups may be explained by the animals’ food which contains proteins. Several studies have revealed that xanthine oxidase is responsible for the formation of uric acid from hypoxanthine or xanthine and is also responsible for the medical condition known as gout. In this sense, the decrease in urea, uric acid, and triglycerides during the treatment might be attributed to the fact that *S. acuta * and *S. cordifolia * have a xanthine oxidase inhibitory property [5].

Concerning the anti-nociceptive activity of extracts from *S. acuta * and *S. cordifolia *; abdominal injection of acetic acid was used to evaluate the extracts on their peripheral analgesic activity [35]. It has been reported that acetic acid irritates the peritoneal cavity leading to stimulation of local nociceptors located at the surface of the peritoneal cavity [36]. This leads to the release of prostaglandins and other algogens with subsequent stimulation of pain nerve endings [37]. Inhibition of the pain induced by acetic acid, by the extracts, suggests that they can probably work by suppressing the release of inflammatory mediators like prostaglandin, bradykinin and histamine [38]. According to the best of our knowledge, there is no data on the analgesic effect of the aqueous acetone extracts from *S. acuta * and *S. cordifolia *. Indeed, the oral administration of the aqueous acetone extracts from *S. acuta * and *S. cordifolia * produced significant inhibition of the acetic acid-induced abdominal writhing in dose-dependent manner. This inhibition was lesser than that produced by paracetamol. These results suggest that extracts from *S. acuta* and *S. cordifolia * can produce peripheral analgesic effect by inhibiting the chemical mediators and/or cytokines. The formalin test is a useful method to evaluate mild analgesic effect of drugs [39]. After formalin injection in hind left paw of mice two distinct phases are observed: the first phase (0 to 5 min) or neurogenic phase comes from chemical stimulation that releases bradykinin and substance P. The second phase, coming 15–30 min after formalin injection, comes from the release of inflammatory mediators such as histamine and prostaglandin [18,40]. The aqueous acetone extracts of *S. acuta * and *S. cordifolia * have inhibited both phases, but the inhibition was more significant in the case of the late phase. Previous studies showed that analgesic activity can result from high flavonoids content as well as from free radical scavenging activity. Free radicals are involved in pain stimulation and antioxidants are known to inhibit such pains [41]. Moreover, recently discovered substances that have analgesic properties includes alkaloids, flavonoids and terpenoids compounds [42]. The presence of flavonoids in the extracts of *S. acuta * and *S. cordifolia *[5] can justify the analgesic activity of the extracts.

## Conclusion

The very weak toxicity values are interesting features which can securize the use of *Sida acuta* and *Sida cordifolia * in the traditional medicine of Burkina Faso. The low toxicity, evidenced by LD50 values, suggests a wide margin of safety for therapeutic doses. The extracts have no significant negative effect on biological parameters. Moreover we obtained good analgesic results by testing the two species aqueous acetone extracts against animal model. Both findings justify the therapeutic use of these plants in folk medicine of Burkina Faso. There still remains the need for elucidating the molecular structures and the precise pharmacology of the active principles.

## Competing interests

The authors declare that they have no competing interests.

## Authors’ contributions

KK and RRAS carried out the experiments and wrote the manuscript, IHNB, AH and AS supervised the work and the manuscript. NB, MHD, JYD and BMB contributed to the manuscript corrections. All authors read and approved the final manuscript.

## Pre-publication history

The pre-publication history for this paper can be accessed here:

http://www.biomedcentral.com/1472-6882/12/120/prepub
